# Predictive systems ecology

**DOI:** 10.1098/rspb.2013.1452

**Published:** 2013-11-22

**Authors:** Matthew R. Evans, Mike Bithell, Stephen J. Cornell, Sasha R. X. Dall, Sandra Díaz, Stephen Emmott, Bruno Ernande, Volker Grimm, David J. Hodgson, Simon L. Lewis, Georgina M. Mace, Michael Morecroft, Aristides Moustakas, Eugene Murphy, Tim Newbold, K. J. Norris, Owen Petchey, Matthew Smith, Justin M. J. Travis, Tim G. Benton

**Affiliations:** 1 School of Biological and Chemical Sciences, Queen Mary University of London, Mile End Road, London E1 4NS, UK; 2 Department of Geography, University of Cambridge, Downing Place, Cambridge CB2 3EN, UK; 3 Institute of Integrative Biology, University of Liverpool, Liverpool L69 7ZB, UK; 4 Centre for Ecology and Conservation, College of Life and Environmental Sciences, University of Exeter, Cornwall Campus TR10 9EZ, UK; 5 Instituto Multidisciplinario de BiologíaVegetal (CONICET-UNC) and FCEFyN, Universidad Nacional de Córdoba, Casilla de Correo 495, Córdoba 5000, Argentina; 6 Computational Science Laboratory, Microsoft Research, 21 Station Road, Cambridge CB1 2FB, UK; 7 IFREMER, Laboratorie Ressources Halieutiques, 150 quai Gambetta, BP 699, Boulogne-sur-Mer 62321, France; 8 Helmhotz Center for Environmental Research, Department of Ecological Modelling, Permoserstrasse 15, Leipzig 04318, Germany; 9 Earth and Biosphere Institute, University of Leeds, Woodhouse Lane, Leeds LS2 9JT, UK; 10 Centre for Biodiversity and Environment Research, Department of Genetics, Evolution and Environment, University College London, Darwin Building, Gower Street, London WC1E 6BT, UK; 11 Natural England, Cromwell House, Andover Road, Winchester SO23 7BT, UK; 12 British Antarctic Survey, Madingley Road, High Cross, Cambridge CB3 0ET, UK; 13 United Nations Environment Programme World Conservation Monitoring Centre, 219 Huntingdon Road, Cambridge CB3 0DL, UK; 14 Centre for Agri-Environmental Research, School of Agriculture, Policy and Development, The University of Reading, Earley Gate, PO Box 237, Reading RG6 6AR, UK; 15 Institute of Evolutionary Biology and Environmental Studies, University of Zurich, Winterhurerstrasse 190, Zurich 8057, Switzerland; 16 Institute of Biological and Environmental Sciences, Zoology Building, Tillydrone Avenue, Aberdeen AB24 2TZ, UK; 17 School of Biology, University of Leeds, Leeds LS2 9JT, UK

**Keywords:** modelling, systems ecology, climate change, ecosystem assessment

## Abstract

Human societies, and their well-being, depend to a significant extent on the state of the ecosystems that surround them. These ecosystems are changing rapidly usually in response to anthropogenic changes in the environment. To determine the likely impact of environmental change on ecosystems and the best ways to manage them, it would be desirable to be able to predict their future states. We present a proposal to develop the paradigm of predictive systems ecology, explicitly to understand and predict the properties and behaviour of ecological systems. We discuss the necessary and desirable features of predictive systems ecology models. There are places where predictive systems ecology is already being practised and we summarize a range of terrestrial and marine examples. Significant challenges remain but we suggest that ecology would benefit both as a scientific discipline and increase its impact in society if it were to embrace the need to become more predictive.

## 1. Background

The importance of ecosystems and their associated biodiversity for humans is well established [1–3]. Yet, our understanding of ecosystem structure and function is far from complete. This is important not just from a scientific perspective, but also because effective protection and management of key ecosystems, and their services, depends on: understanding how they will respond to a range of contemporary pressures; and projecting realistic future scenarios so as to enable decision making. Projecting ecological models into the future is challenging because ecological systems are inherently complex, nonlinear and variable, while future conditions often lie outside the envelope of parameters used to develop models. It is therefore not surprising that confident predictions of ecosystem dynamics are rare, despite being in demand from society. A case in point is the UK National Ecosystem Assessment (NEA) [2] which is one of the most rigorous and systematic assessments of ecosystems and their ability to meet multiple needs of the UK population now and in the future. Despite being based on the unusually rich and complete information available for the UK, its authors had to state:

‘scenarios attempt to look to the future and describe worlds very different from today's, the ecosystem responses have to be credible…. This is generally achieved by using either process-response models or empirical relationships that would allow drivers and ecosystem services to be quantified,… Unfortunately, the UK NEA material on current state and trends provided few models or empirical relationships of the type needed’. [4, p. 1207]

In addition to predicting how ecosystems may respond to environmental changes, models are needed to evaluate potential management options. We therefore need:

— the development of ecological system models that can be used to forecast the possible future state of a system;
— the use of such models to determine the likely impacts of plausible futures, for example climate change, changes in land use patterns, changes in nutrient flows; and
— their further use to test response options designed to mitigate, or adapt to, the impacts of change.

While the ability to predict accurately how a system will respond to perturbations is seen by some as a defining characteristic of a successful science [5], this has not always been a significant focus within ecology [6–15]. We believe that the societal imperative to predict the impacts of environmental change on ecosystems (usually, but not exclusively, the result of anthropogenic pressures) should add impetus to this endeavour [16].

In this paper, we are concerned with *forecasts* of the most likely state that a system may have in the future, this would be one of the possible *projections* for that system—the set of all possible states that a system could have in the future (http://www.ipcc-data.org/ddc_definitions.html). In the language of the Intergovernmental Panel on Climate Change, this would mean that the general process that is required is the development of projections of the future state of ecosystems that will enable us to move towards forecasting the most likely future state.

## 2. What is needed to make a projection?

If we are to predict biological responses to environmental change, we will need models that generate accurate and realistic projections under both present and future, potentially novel, conditions. These requirements mean that two of the conventional approaches to ecological modelling—very simple models containing few parameters; and phenomenological models derived from observed statistical relationships among parameters—are unlikely to be reliable [17]. The former are so removed from real-life systems that they can rarely be meaningfully tested against data, while phenomenological descriptions of data should not be used to extrapolate beyond existing conditions [18–20], hence cannot reliably be used to predict responses to novel conditions. To predict biological responses to environmental change, we need *process-based* ecological models that capture the important underlying biological mechanisms driving the behaviour of the system [21–24], akin to systems models used in climatology [25,26] and molecular systems biology [27].

Predictions of ecosystem behaviour (the way in which the properties in an ecosystem change over time or in response to perturbation) and/or ecosystem services (which are products of ecosystems and often are valuable to society) would be emergent properties of process-based models of ecosystems [28], and such models would need to include sufficient information from lower levels of organization (populations, individuals, genes and the abiotic environment) to allow accurate and realistic ecosystem behaviour to emerge [16].

## 3. Previous systems approaches in ecology were unsuccessful

An integrated ecological systems approach was attempted in the 1960s [29,30], most notably in the International Biological Programme (IBP) [31]. The systems ecology of the 1960s foundered on at least two grounds: practically—the computing power available in the 1960s was insufficient to run the necessary models; and philosophically—the approach attempted to measure as many parameters of a system as possible without using theory beyond its use as a tool for informing which function might best fit a given dataset [32,33]. Our use of the term ‘systems ecology’ is distinct from the approach used in the IBP, instead it is analogous to the approach used in climatology and in systems biology where process-based models are derived from observational and experimental data.

Recent technological advances are revolutionizing scientific research. New sensors allow us to collect data in novel ways: individuals can be tracked in the wild; ecosystem services or habitats can be mapped remotely. New informatics tools permit better data collation, analysis, inference and visualization. Formerly undreamed-of computing power is now ubiquitous. Additionally, there have been advances in theory: in evolutionary ecology [34]; behavioural ecology [35]; and life-history evolution [36]. These disciplines, which emerged from the adaptationist paradigm [37] in the 1970s and 1980s, focused on providing explanations for individual behaviour derived from first principles and concerned with evolutionary function. These theories can provide explanations for why organisms act as they do. Recent work has provided rigorous explanations for why we would expect individual organisms to maximize relative fitness [38,39]. If we accept this, and if we have a good understanding of how to measure fitness [38], then we have a conceptual basis for modelling life-history decisions of organisms, even when we have sparse information about their biology and potentially even in conditions that we have not observed previously. For an example in which individual foraging decisions (behavioural ecology) were successfully used to predict food web complexity (community ecology), see [40]. Moreover, the development of individual-based and spatially explicit modelling provides tools to model ecological systems that integrate the behaviour of individuals [41] to their life histories and allow phenomena at higher levels of organization (populations, communities and ecosystems) to emerge from the interaction of processes at lower levels of organization [7,10] and spatial extent [42].

## 4. A new kind of systems ecology

We believe that the time has therefore come to develop ‘predictive systems ecology’ which we define here as ‘the integrated analysis of interactions and feedbacks across different components of biological and ecological organization and scale, and their relationships with their abiotic and biotic environments, to understand and predict the properties and behaviour of ecological systems’.

The societal imperative of managing systems under change should facilitate the development of a fundamental scientific good: systems approaches that lead to understanding. Prediction and understanding have often before been considered as separate modelling goals within ecology: models are often typified as strategic (leading to understanding) or tactical (allowing prediction) [24,43,44]. However, we consider this a false and potentially damaging dichotomy. Process-based models can be developed with the dual aim of improving both mechanistic understanding and predictive capability [45]. We will have the basis for understanding an ecological system if we can make predictions about its state in new conditions. Indeed, ecological theory will only be scientifically credible when its predictions can survive being comprehensively testing against the widest possible range of data.

The pace of change—in technology, in computing and in the environment—is enormous. This sets the scope of both the challenge and the potential of meeting it. This potential can arise from the bottom-up embracing of the requirement, and developing opportunities, for doing ecology in a new way, and it can be driven by top down social imperatives.

## 5. Considerations for systems approaches

### (a) Uncertainty

Ecological system models have to embrace uncertainty to an even greater extent than either climatology or molecular systems biology, because at the core of climatological models are well-understood physical equations, while in systems biology predictive ability is enhanced by the deterministic nature of chemical interactions between populations of molecules [27]. Systems ecology has to grapple with uncertainty as, for example, stochastic effects occur throughout ecological systems at all levels [46]. The propagation of stochastic effects may mean that confidence intervals on projections will be large but realistic measures of our uncertainty, reflecting our ability to predict outcomes in the real world given our current state of knowledge [47]. We will increasingly require ensembles of model runs with different initial conditions, parameter sets, or even processes (cf. [48]) in order both to scope out the variability of outcomes, and to quantify context-dependent predictability of future states. This approach is routinely followed in weather forecasting [49], and similar needs apply to ecological forecasting.

The sources of uncertainty in the modelling process need classification [50–53]. At the very least, there are uncertainties in model parameters, in the specification of processes that are included, and in the datasets that inform the models; there will be processes that we know to be operating in principle, but about which we lack enough knowledge, or data, to make sensible model specifications, and there will be real-world processes about which we are currently ignorant. Additionally, it can be unclear where to draw model boundaries, either spatially, or temporally, or in terms of what processes and categories of object should be included, and at what resolution and scale the model should be run. Examples would be: cohort-based models that produce different outputs from models that include all individuals in a population [54]; and patchiness and variability affecting productivity and ecological dynamics [55,56]. Finally, there may be causal uncertainty: in a complex system, the apparent proximal triggering of an event may not be the main dynamical reason for its occurrence. Indeed, there may be no one single factor that can be identified as leading to a given outcome. Predictive systems ecology will need to find new ways of formalizing both the treatment of stochasticity and the communication of uncertainty [57].

### (b) Complexity

Predictive systems ecology is ‘big science’. It requires large amounts of data and complex models. To avoid the fate of classical systems ecology, model complexity must be decided carefully, and standardized approaches for describing individual organisms and their interactions and model structure are required. Just as big data analysis requires advanced computational statistics, predictive systems ecology will require models as complex as necessary to realistically represent ecosystems. In a complex system with multiple patterns, there may be trade-offs between agreement with different datasets or advantages in some cases to including particular processes that are not required for others. In such cases, a multi-model approach with replaceable components allows for different model purposes to be accommodated within an overarching scheme [58]. Exploring experimental model variants can provide the level of complexity merited by iteratively removing or adding components to the model and repeatedly comparing model robustness and sensitivity with data [59]. This component-based approach would allow for suites of models to be developed with different targets in mind that have different levels of internal complexity depending on the state of knowledge for particular subsystems, and is attractive from the software development point of view, as it allows for flexibility in model development and implementation, so that different groups of specialists can develop their own component parts independently.

The computational burden of predictive systems ecological models is not to be underestimated, but the judicious use of mathematics can mitigate this by highlighting approximations that can replace some components of explicit simulation models. For example, it is more computationally expensive to model the motion of organisms by discrete steps than to use the diffusion equation, yet in some cases the latter may describe the behaviour equally faithfully [60]. Moreover, the diffusion equation is more parsimonious as it requires just one parameter (the diffusion constant) to be measured, whereas explicit simulation requires many details. In many cases, mathematical approximations work best in exactly the cases where simulation is most costly, e.g. when local density dependence is made up of contributions from many individuals [61]. By incorporating, at the right scale, appropriate analytical and semi-analytical submodels into simulation approaches, we can simulate more complex ecological systems and improve the power of our projections.

The high levels of uncertainty and complexity in ecological systems will be seen, by some, as a reason not to attempt progress. We suggest the opposite: that these create interesting opportunities for the development of new theory—perhaps furthering fruitful collaborations between ecologists, statisticians, mathematicians and computer scientists. We should also note that error does not necessarily multiply in process-based models of complex systems: there can be feedbacks and buffers that reduce the effects of variation, just as in real systems. Such models would allow us to see effects that at present are not conspicuous. Complexity despite being inherent in understanding ecology as the product of its component parts, often remains untackled while researchers focus on simplifying systems to make them more tractable [62].

### (c) Constraining models with data

Greater emphasis on constraining models, with data, and on hypothesis testing within models will afford both more robust predictions to be made from models and better understanding of the processes that are likely to be operating within ecosystems. Models should be expected to generate projections at many different levels—both within the ecosystem and emerging from the system; these could and should be tested against data. Testing such patterns is a core concept of predictive systems ecology. The very fact that we can observe patterns means that in ecology, despite of all the uncertainty, nonlinearity and stochasticity, there are recurrent phenomena, and there is something to be predicted. Models should be able to reproduce these observed patterns, both qualitatively and quantitatively [63]. Still we may have to accept that high process accuracy may not, in all cases, lead to high agreement between model and past observation [64], and that multiple process pathways can potentially give rise to similar output patterns, so that model selection may not be straightforward [65].

Systems ecology will need to develop methods to deal with the availability of data—a significant advance could be made in ecology if more scientists working in the discipline adopted the habit of data sharing (www.datadryad.org), and funders made free data access a condition for funding, as is the norm in some in other areas of science, and is being actively encouraged by funders in most countries. Successful models will also suggest key gaps in data and hopefully inspire new and relevant observations and experiments. Common standards will be needed for both data and models, to make both more transparent and easily handled [66]. This has been done elsewhere: for example systems biology markup language facilitates model interoperability and sharing and the existence of publically funded data repositories makes data sharing relatively straightforward in molecular biology.

## 6. Systems ecology is already being practised

Systems ecology approaches are already being applied in both terrestrial and marine systems.

### (a) Terrestrial vegetation models

Terrestrial vegetation models have been developed, at least in part, to refine the terrestrial carbon cycle subroutines of general circulation models and the Earth System Models, and to improve the predictive ability of these models [67]. Models like SORTIE, FORMIND, PPA, PICUS and ED are capable of producing robust predictions of community structure in forest ecosystems over time [21–23,68–71]. In the main, this field has developed from a combination of ecophysiology and individual-based modelling, as well as data from established, long-term, forest inventory surveys, allowing model output to be tested against observations. The rationale for the development of these models has been to improve predictions of how ecosystems respond to changes in climate and, in particular, how these changes will feedback into and affect the global and regional climate [67]. The development of land process models has burgeoned, with each model incorporating the same fundamental processes to predict the same global phenomena, such as the dynamics of terrestrial carbon. Now, the focus is on why the different models make different predictions which are resulting in model-intercomparison [72–74], benchmarking models against standard datasets [75,76], model–data fusion [77,78], and characterizing the expected behaviour of the terrestrial carbon cycle [79]. The development of terrestrial vegetation models has benefited from the existence of, often rich, datasets collected by foresters in many parts of the world. These allow the growth, development and reproduction of trees to be well constrained by data and the increasing use of Bayesian methods to infer probability distributions for model parameters allows estimation of the likely error in both parameters and output (for a recent example, see [78]). Terrestrial vegetation models that are based on modelling the behaviour of individual trees (e.g. SORTIE and FORMIND) can be computationally demanding when extended over large areas and a solution to the problem of scaling these models up has been to move from individual-based to cohort-based models (e.g. ED is a cohort-based model). A version of ED parametrized with data from a Harvard Forest, MA, has been used to generate predictions of forest structure across a large area of the northeastern USA and southeastern Canada that are a good match to what is seen in forest inventories [71]. The solution to the inherent practical difficulty of scaling up to larger areas was to lose information about the behaviour and performance of individual trees.

The main focus of terrestrial vegetation models is on primary producers and their relationship with the climate via the carbon cycle [67], effectively this relationship is at the core of the climate-regulating services provided by ecosystems. While some other ecosystem goods and services could be derived from terrestrial vegetation models—food, fibre and biofuels—the limited scope of these models means that estimates of most ecosystem services cannot be derived from them.

The output from terrestrial vegetation models could be converted into estimates of primary production and thence to the abundance and distribution of resources that could then be used to model herbivore behaviour. The impact of herbivory would impact on the plants and could feedback into the vegetation model. There has been some consideration of the impact of herbivorous insects on tree mortality, growth and carbon capture in forest models [80,81]. Neither of these studies was concerned with estimating herbivore populations but could be modified to do so, taking forest models closer to models of ecosystems.

### (b) Ocean ecosystem models

A particular focus of ocean ecosystem modelling has been on models of biogeochemical cycles, developed in conjunction with the ocean physics and chemistry modelling communities. These ocean ecosystem models are process-based, but the focus is on physical and chemical physiological processes. There are models of microbial systems in which organisms have been categorized on the basis of the different groups with different biogeochemical functions. Examples of such models include MEDUSA, that has relatively few components but is physiologically complicated [82], and PlankTOM10, which includes a wider range of functional types [83]. These models are being used to examine the role of these systems in global biogeochemistry, and especially carbon budgets.

None of these marine models were designed to examine the ecological impact of environmental change or to act as a model of the ecosystem as a whole. Models have been developed to examine individual species in oceanic systems. These have developed over the last decade to be coupled with output from physical models to examine the interaction between biological processes (physiological, behavioural and demographic) and physical processes (e.g. ocean circulation and vertical structure [84–88]). In one global marine phytoplankton model, physiological traits were assigned randomly to generate different phytoplankton types. This model generated a realistic emergent community structure at a global scale [89].

A range of marine food web models have been developed to examine the impacts of change within ecosystems; including impacts of fisheries and climate change. However, these make various simplifying assumptions to cope with the inherent complexity such as aggregating across trophic levels and averaging processes across spatial and temporal scales (e.g. ECOPATH [90–92]). There have been other approaches to capture the broader structure of ecosystems, with a particular focus on development of size-based models (e.g. APECOSM [93,94]), which again make simplifying assumptions to cope with the complexity of having many different species. However, the importance of cross scale interactions and feedback processes is now being recognized: for example, plankton and fish populations are affected by the biogeochemical and physical systems dynamics and large zooplankton species may influence biogeochemical cycles [95]. The challenge of generating climate change projections has led to the development of systems level concepts that in the marine community have been termed ‘end-to-end’ approaches [96]. This includes the development of methods for linking together different types of models such as biogeochemical, population and food web models [97]. A key aspect of this work is to link to human activities, focusing mainly on fisheries activities, and a range of models of food webs and fisheries are being linked to biogeochemical and physical models [98,99]. Some key features of ocean ecosystem models are that they frequently operate at large scales (sometimes a global scale) and that they integrate the physical environment into models more extensively than is typical for terrestrial models. The low availability of datasets for many parts of the world's oceans can limit the ability to constrain these models with data (for a discussion of this issue in the Southern Ocean, see [97]).

### (c) Global ecological models

A prototype global ecological model has been recently reported [100], building on the idea of enabling the structure and dynamics of ecological systems to emerge, at global scales, from the fundamental birth, death, interaction and dispersal dynamics of modelled individuals. This model uses simplifying assumptions such as representing organisms as cohorts of identical individuals rather than individuals *per se* and uses functional types rather than species, but even in its current, initial instantiation, is capable of generating simple but realistic predictions at the level of the entire biosphere from rules about the behaviour of individuals (birth, death, growth, dispersal and interactions). This model, rather like the terrestrial vegetation model ED, makes a series of simplifying assumptions in order to overcome the obvious practical difficulties of trying to model all individuals in a large area. This is clearly necessary to make the problem tractable but does result in the loss of information about the finer detail—providing a good example of the trade-off between the detail that it is possible to capture in a model and the scale at which it operates. This might mean that, for example, it was difficult to see how evolutionary change could be accommodated in such a model.

### (d) Humans and ecosystems

People interact with ecosystems, and almost all systems have been modified to some extent by human intervention [101] and many are dominated by human activity. There are a growing number of examples of coupled models of humans and ecosystems, such as Bithell & Brasington [102] who coupled SORTIE with a hydrological model and an individual-based model of a human population. This model was used to predict the extent of forest clearance over a 1000-year timescale in a mountain valley. The human population expanded, clearing trees for fuel and to produce fields. The model suggested that the loss of forest cover and the increase in crops would result in changes in the hydrology with a tendency to greater flash flooding during the monsoon. This coupled model is an ecosystem model with several types of plant (two types of trees as well as food crops), a ‘herbivore’ (humans) and the hydrology of the environment in which they reside. This model was able to produce a reasonable description of current state of the valley and forecast valley state into the future [102]. Other studies have looked at landscape evolution in coupled models of herbivores, hunter–gatherers and vegetation [103], populations of migratory wildebeest, pastoralists and vegetation [104], bushmeat hunting [105], and have examined management options for pastoralists in rangeland systems [106]. Models for marine systems have included aspects of the dynamics and/or economics of fishery fleets that have been linked to models of fish populations and oceanic ecosystems [98,107–109]. These exercises suggest that producing coupled human–ecosystem models that capture key elements in a food chain is achievable. They also provide good examples of models that include the effect of people on ecosystems.

Understanding how human behaviour and policies might respond to the loss of natural resources and compensate for this loss, will require linked, dynamic human–ecological systems models [110–112], further adding to the challenge of building truly predictive ecosystem models. The feedback between humans and ecosystems will need to be a feature of systems ecology models, not just because policy makers and legislators are more interested in the impact of change on people than on ecosystems, but also because in many cases the activities of humans have larger effects on ecosystems than those of any other component of the ecosystem [113]. Very significant challenges remain in approaching a full ‘social–ecological–system’ framework for coping with the interactions between humans and the environment [65,114], not least the human ability to self-organize in different ways at different institutional levels [115]. However, the likelihood is that few if any unmodified ecosystems remain against which a ‘pristine’ ecosystem model could be adequately tested and so understanding human-modified systems will be unavoidable.

## 7. What are the challenges?

### (a) Scale

Different biological processes operate over different scales and interact with different physical and chemical processes [116,117]; so ecosystem models require the application of scaling rules, both physical and ecological, from local and regional to global scales and across different levels of biological organization and processes (gene, individual, population, community, food webs and ecosystems) [28,95,97]. Developing models that resolve the appropriate physical, chemical, biological and social processes at different scales presents a major challenge [99,118–121], but scaling from individual behaviours to changes in population sizes at a regional scale is being attempted [100,122].

### (b) Evolution

Evolutionary change is also a ubiquitous feature of living systems. The extent to which it needs to be incorporated in ecological models will be determined by the relationship between the duration over which projections are made and the generation times of the organisms of interest. While models of forests typically run for periods equivalent to centuries or even millennia, the generation time of the trees will mean that only small number of generations occur, and so evolutionary change during that time is assumed to be sufficiently small to be practically negligible [22,70]. However, were one to incorporate trophic interactions with univoltine forest insects, then evolutionary change, particularly in a changing environment, becomes likely. If we truly wish to understand biological diversity and function then it is not appropriate to treat the ‘evolutionary play’ as being distinct from the ‘ecological theatre’ in which it occurs [11–13,34,123]. Ecological and evolutionary change are intertwined—population dynamics are the product of the realized life histories of individuals within the population, while the strength of selection is modified by properties of the population [13,34,124,125]. So far, there have been few attempts to include evolution into ecological models (examples are available for fisheries-induced evolution [110,111], and for freshwater [126] and terrestrial ecosystems [127]) and this scarcity is needs to be rectified [125,128–130].

Recognizing the importance of evolution also helps us to model organisms with poorly known behaviour. If we know that organisms will act so as to maximize relative fitness [38,39] then we have a conceptual basis for modelling life-history decisions of organisms, even when we have sparse information about their biology [40]. Understanding evolutionary change will also become critical when we are faced with rapid environmental change and consequently intense selection—evolution then potentially becomes the primary process in shaping the future of ecosystems. This will have substantial effects if some components of ecosystems fail to adapt and are lost during the process of change [131]. This may happen when multiple environmental variables change simultaneously and rapidly, such as is often true for anthropogenic changes.

## 8. Conclusion

The need for a modern approach to systems ecology is clear, the requisite theory and data exist at least to start the process, and the societal imperative should provide impetus for the development of this field. As we transgress our local and planetary boundaries [132], the need for better understanding of the world across multiple space and time scales becomes ever more urgent. Process-based models should be the best available tools we have to in hand in this struggle: they will help us to distinguish those systems that are dynamic, contingent, threshold-dependent and changing from those that are stable and resilient; to separate situations in which robust forecasts can be made from those that are chaotic or indeterminate and to elucidate those cases that are amenable to simple explanation and identify those that are irredeemably complex. Along this path, the act of developing and constructing new models, in which assumptions about ecosystem dynamics must be made explicit and shown to be operational, has multiple potential benefits [133], not least to identify theoretical and data collection gaps and opportunities, and to confront existing ideas with new datasets in a meaningful way. With a suite of these models, we might hope to get to grips with the multiple issues of policy relevance that ecosystems present [134], although there will remain many difficulties in the translation from model to policy [135,136]. However, relevance to policy should not be the sole driver of our interest: developing predictive systems ecology should also inspire us through new and surprising discoveries. To truly forecast the future of Darwin's ‘.. tangled bank, clothed with many plants of many kinds, with birds singing on the bushes, with various insects flitting about, and with worms crawling through the damp Earth’ [137, p. 403], we must embrace fully the complexity of the natural world and include it in our models.
